# Food Bank Donations in the United States: A Landscape Review of Federal Policies

**DOI:** 10.3390/nu12123764

**Published:** 2020-12-08

**Authors:** Katelin M. Hudak, Emily Friedman, Joelle Johnson, Sara E. Benjamin-Neelon

**Affiliations:** 1 Department of Health, Behavior and Society, Johns Hopkins Bloomberg School of Public Health, Baltimore, MD 21205, USA; sara.neelon@jhu.edu; 2 Center for Science in the Public Interest, Washington, DC 20005, USA; efriedman@cspinet.org (E.F.); jjohnson@cspinet.org (J.J.); 3 Lerner Center for Public Health Promotion, Johns Hopkins Bloomberg School of Public Health, Baltimore, MD 21205, USA

**Keywords:** emergency food, food insecurity, food security, legislation, regulations, statutes, nutrition, healthy

## Abstract

Rates of food insecurity have increased substantially in the United States (US), and more families are turning to the charitable food system to help meet their needs. Prior studies have examined the nutritional quality of foods offered through food banks, but little is known about what government policies may shape the healthy food donation landscape. The purpose of this study was to review US federal policies that impact food and beverage donations to food banks and assess whether policies encourage healthy food donations. In spring 2020, two researchers independently reviewed federal food and beverage donation policies using predefined search terms in two legal databases. We identified six categories of policies based on the existing food donation literature and themes that emerged in the policy review. We identified 42 federal policies spanning six categories that addressed food and beverage donations to food banks. The largest category was “government programs,” with 19 (45%) policies. The next largest category was “donation via schools,” with 12 (29%) policies. However, no policies specifically addressed the nutritional quality of food donations. There is an opportunity for the federal government to strengthen food bank donation policies and improve the nutritional quality of donated foods and beverages.

## 1. Introduction

Coronavirus disease 2019 (COVID-19) has created a global public health and economic crisis. As a result, rates of food insecurity rose dramatically in 2020, with one in ten (10.1%) adults in the US experiencing food insecurity in September [1]. Food insecurity was even higher among households with young children; 40.9% of households with children ages 12 and under reported experiencing food insecurity since the beginning of the COVID-19 pandemic in 2020, which was a 170% increase from 2018 [2]. As the United States (US) continues to cope with high unemployment and economic uncertainty, rates of food insecurity are expected to increase further. Federal food assistance programs such as the Supplemental Nutrition Assistance Program (SNAP) are a key aspect of the social safety net that are designed to alleviate hunger and help families afford a nutritious diet [3]. However, SNAP benefits may not be large enough to help households adequately meet their dietary needs [4]. The charitable food system fills the gap left by SNAP and other food assistance programs. In response to the pandemic, families are turning to the charitable food system in greater numbers to help meet their needs. Of the 200 food banks in Feeding America’s national food bank network, 98% reported an increased demand for food assistance, and 59% reported having less inventory since the beginning of the COVID-19 crisis in March [5]. Ariel footage of a miles-long line of cars waiting to receive food from the Greater Pittsburgh Community Food Bank in April provided a stark picture of the surge in demand [6].

Studies on the nutritional quality of foods and beverages distributed by food banks have emerged primarily in the past decade. A 2018 report by MAZON and the Rudd Center found that nearly one-third of food distributed by 196 surveyed food banks was fresh produce [7]. However, a review of inventory at six California food banks found that approximately half of the fruits and vegetables were potatoes and onions, which are less nutrient dense than many other types of fruits and vegetables [8]. In addition, a 2017 national survey of food banks found that approximately one-quarter of distributed foods and beverages were high in added sugars, sodium, and saturated fat [7]. This finding is especially important, as low-income families who are more likely to utilize emergency food sources have an increased risk of diet-related chronic diseases, such as heart disease, stroke, and type 2 diabetes [9,10].

The federal government has implemented policies impacting food donation over the last several decades, often in response to disasters or economic downturns that drive more people to use the charitable food system: for example, The Emergency Food Assistance Program’s (TEFAP) establishment in 1983 was in part a response to a recession [11] and the now-permanent Enhanced Federal Tax Deduction for Food Inventory was initially a temporary measure in response to Hurricane Katrina [12].

A key study by Shimada et al. [13] laid out a conceptual framework of stakeholder and policy influences on the nutritional quality of emergency foods. Study authors illustrated that the nutritional quality of emergency foods is multi-layered and shaped by economic, political, and social factors. Clients’ food preferences and nutritional needs are at the center of their framework [13]. Moreover, recent work by the Harvard Food Law and Policy Clinic, the Natural Resources Defense Council, ReFED, and the Center for EcoTechnology reported on policies that may influence the quantity of foods that enter the food bank system [14,15,16,17,18,19,20]. However, little is known about whether existing policies promote healthy food bank donations. Therefore, the purpose of this exploratory study was to review federal policies regarding food bank donations and assess whether any policies included language encouraging healthy food donations. Given the lack of research in this area, we consider this study to be hypothesis generating rather than hypothesis testing.

## 2. Materials and Methods

### 2.1. Study Design and Overview

For this cross-sectional policy study, we reviewed current federal policies (e.g., statutes, legislation, regulations, and administrative decisions) related to food bank donations from March to June 2020. We used two legal databases—Westlaw and LexisNexis—to identify policies using a predefined Boolean search string [21]. (The search strings we used were (food-bank food-pantr* glean** food-insecur*** “food donations” “donor of food”) & (donat**** donor) & food (Westlaw) and ((food bank! or food pantr! or glean! or food insecur! or “donated foods” or “donor of food”) and (donat! or donor)) and food (LexisNexis).) We included search terms to capture policies related to food banks, food pantries, food donations, and gleaning. (Gleaning refers to the act of collecting leftover fresh foods from farms, gardens, and other sources in order to give it to those in need [22].) The Johns Hopkins Bloomberg School of Public Health Institutional Review Board did not require ethical review because human subjects were not included in this policy review.

### 2.2. Inclusion/Exclusion Criteria

We included policies that could potentially influence the quantity or quality of foods and beverages donated to food banks. Relevant policies included those related to offering tax incentives, liability protection, or government grants that food banks can use to support their donation programs. We excluded general grants that are available to food banks, e.g., grants that food banks can use to cover administrative or infrastructure expenses. We also included other policies encouraging donations directly from a federal program, such as United States Department of Agriculture (USDA) commodity-purchasing programs, or policies that authorized the donation of specific foods (e.g., wild game). We excluded policies that were no longer good law (i.e., laws that were repealed or expired) or did not mention food bank donations, or were focused on food or beverage donations outside of the US. We further excluded case law, secondary sources, and legislative histories.

### 2.3. Review of Policies

Two independent and trained reviewers conducted the searches using online legal databases; one used Westlaw and the other LexisNexis to conduct the searches. Reviewers compared results to determine which policies were identified and included in both searches, and which policies were identified and included in one search but not the other. Reviewers resolved any differences via a team discussion. Because reviewers used two different databases, the majority (97.2%) of discrepancies among policies were due to the policy appearing in one but not both legal databases. (The remaining 2.8% of differences were due to questions of whether a policy fit the inclusion criteria, e.g., a grant that was more general in nature, such as for community food projects, e.g., 7 U.S.C.A. § 2034. (This policy was excluded).)

We identified 37 relevant policies from the two databases: federal laws, regulations, or administrative guidance that affect food and beverage donation to food banks. Reviewers then conducted targeted online searches for policies that were not identified via the Boolean search string and identified an additional five policies for a total of 42 federal policies related to food bank donations in the US. (The five policies that the search string did not identify were related to child nutrition program meal patterns or to policies enacted in response to COVID-19; it is unclear why the search string did not identify these policies. Reviewers were aware that these policies existed, and therefore conducted targeted searchers for them.) We then sorted policies into six broad categories based on existing literature and resources on food bank donations, and patterns that emerged as we reviewed the policies: [14,15,16,17,18,19] liability protection, tax incentives, donation of certain food(s), donation via schools, grant program or fund, and government programs (Table 1). Some policies fell into more than one category; almost all policies that fit into multiple categories were Farm Bills (Table 2). We conducted frequencies for each category using Stata 16.1 [23]. We then reviewed each policy qualitatively and assessed whether the policy explicitly or in practice promoted the donation of nutrient-rich foods, consistent with our approach in prior policy reviews [24,25,26]. Rather than using an external definition of “healthy,” our aim was to document any policies that referred to healthier foods or beverages or attempted to promote the donation of them.

## 3. Results

### 3.1. Policy Categories

Of the 42 federal policies we identified, 19 were regulations, 20 were statutes and legislation, and three were administrative decisions. Supplementary Table S1 lists each policy and relevant text. As noted, policies could fall into multiple categories. For example, the Agriculture Improvement Act of 2018 (115 P.L. 334), colloquially known as the 2018 Farm Bill, includes statutes affecting the Dairy Product Donation Program (7 U.S.C. § 9071) and pilot projects to encourage the use of public-private partnerships committed to addressing food insecurity (7 U.S.C. § 2036d). Thus, we categorized 7 U.S.C. § 9071 as “government programs” and 7 U.S.C. § 2036d as “grant program or fund.”

The largest category was government programs, with 19 (45%) policies (Table 2). Example policies are the Dairy Product Donation Program (7 U.S.C. § 9071) and the Federal Food Donation Act of 2008 (42 U.S.C. § 1792). The next largest category was donation via schools, with 12 (29%) policies falling into this category. Donation via schools includes policies in which surplus program foods (e.g., National School Lunch Program/School Breakfast Program) are donated directly to food banks. Program foods are either USDA foods (i.e., purchased as part of a price-support system [27]) or food that schools purchase with USDA cash reimbursements. Seven (17%) policies fell into the category donation of certain food(s). For example, the Prohibited Species Donation Program (50 C.F.R. § 679.26) allows for the donation of salmon and halibut when caught in a protected area off the coast of Alaska. Five (12%) policies were grant programs or funds. These policies authorized grant programs to support healthier food banking projects. For example, food banks may apply for emergency food program infrastructure grants (7 U.S.C. § 7511a in 110 P.L. 246), which can be used to support improved capacity for perishable foods and a more diverse donation pool from local producers. Three (7%) policies provided tax breaks for the donation of food to food banks. 26 U.S.C. § 170(3)(C) makes corporations eligible for tax deductions if they donate food inventory and 26 C.F.R. § 1.170A-4A outlines the regulatory details. The smallest category was liability protection; two (5%) policies protected the liability of food donors. A key policy in this category is the Bill Emerson Good Samaritan Food Donation Act (Emerson Act) (42 U.S.C. § 1791), which protects both the person who donates and the distributing organization from civil or criminal liability, as long as the food is apparently fit when it is donated and any injury of the recipient is not due to gross negligence or intentional misconduct.

### 3.2. Promotion of Healthy Foods and Beverages

Next, we assessed each policy and considered whether it promoted the donation of healthy foods and beverages. A few policies encouraged the donation of certain foods that may be nutrient-rich (e.g., traditional native foods [25 U.S.C. § 1685 in 113 P.L. 79] or salmon or halibut [50 C.F.R. § 679.26]). In addition, foods donated from school meals programs will, by default, fit the same nutrition standards to which school meals are subject [28]. Similarly, USDA-purchased and donated commodities will fit the nutrition standards of the particular nutrition program under which they are provided to food banks. However, the nutritional quality of donated foods was a secondary consequence of these policies and not included in the original purpose. Thus, we did not identify any policies that explicitly promoted healthy food or beverage donations.

## 4. Discussion

In this cross-sectional policy review, we identified 42 federal policies related to food bank donations. A large number of policies (19) were related to “government programs”, followed by 12 policies that fell within the “donation via schools” category. However, no policies directly addressed the nutritional quality of food donations or encouraged healthy food and beverage donations. Instead, current policies are aimed more at shielding donors from liability if donated food harms recipients (e.g., liability protections) or increasing the quantity of donations.

The lack of policies encouraging healthy food donations is concerning—especially as rates of food insecurity continue to rise in the US. Low income and race/ethnicity have been associated with food insecurity [29,30]. Black or Latino households were more than twice as likely as white households to experience food insecurity in October 2020 [1]. Furthermore, families served by the charitable food system are often more at risk of diet-related chronic diseases [9,10]. Poor diet has been linked to 13.2% of cardiovascular disease mortality [31,32], and income is a key predictor of diet quality [33,34]. In the period 2015–2016, nearly 60% of adults with an income-to-poverty ratio < 1.30 had poor diets, whereas less than 40% of adults with an income-to-poverty ratio ≥ 3.0 had a poor diet, as defined by the American Heart Association diet score [31]. As such, the charitable food system is positioned to play a unique and growing role in improving the disparities in diet quality and chronic disease risk that exist along socioeconomic lines. Moreover, evidence suggests that families who use food banks prefer nutrient-rich foods [35,36,37]. Campbell et al. found that New York food bank clients would rather receive meat, poultry, fish, vegetables, and fruit over candy, soda, and snack food [35]. A 2019 Natural Resource Defense Council-commissioned survey of food pantry clients in Baltimore found that 24% were somewhat or very unsatisfied with the amount of produce and protein available to them, and would prefer to receive more of these types of foods [37]. These findings exemplify two of the seven deadly “ins” of charitable food and beverage donations—inappropriateness and nutritional inadequacy—that sociologist Janet Poppendieck discusses in her book *Sweet Charity?* [38] She points out that the charitable food system is driven by supply, rather than client need or preferences. Less healthy foods and beverages such as processed snack foods and candy are produced en masse; what is not sold in grocery stores is likely to be donated to food banks [38]. As such, the nutritional needs of emergency food system clients are often not a factor in determining which foods are donated to food banks [38].

That donations that come from various sources likely shape the nutritional quality of food in the emergency food system. The three largest sources make up 70% of all donated food [39]. Donations from the retail food sector comprise the majority of donations, comprising one-third of the 3.6 billion meals donated to the Feeding America food bank network in 2019 [39]. Federal commodities provided 1 billion (23%) meals, and fresh produce from farmers provided 622 million (14%) meals [39]. Federal commodities and fresh produce are generally nutritious foods. Fresh fruit and vegetables are nutrient-rich foods that are linked with a lower risk of major chronic diseases [40,41,42]. Federal commodities provided through TEFAP received a score of 88.9 on the Healthy Eating Index (the Healthy Eating Index is a measure of overall diet quality that has a maximum score of 100, where higher numbers indicate better diet quality [43]) in 2012, which is higher than the average American diet score of 57.5 [43,44]. Thus, foods from the second- and third-highest categories (federal commodities and fresh produce) are likely to be more nutritious. However, the types of foods donated from the retail sector vary. According to a 2012 report by the Food Marketing Institute and Feeding America, 99% of national retailers surveyed donated general grocery items (e.g., dry goods), and over half (57%) donated produce [45]. However, retailers also donated less nutritious foods. For example, 64% of retailers donated bakery goods, which could include bread, but also cakes and sweets [45]. It is important to note that these figures represent the percentage of retailers donating food in a given category; information on the quantity of food donated in each category is unavailable. Pounds donated is the standard tracking metric, and measure of success, for retail donation programs [45]; this metric privileges the quantity of donations over the nutritional quality of donations.

One challenge of crafting policies to incentivize the donation of nutritious foods and beverages is defining what is “healthy.” In her book *Food Politics*, Marion Nestle points out that defining what constitutes nutritious food is complex because of the politicized nature of federal nutrition guidelines, among other factors [46]. Even so, there have been efforts to come up with a definition of “healthy” that can be practically applied. In 2019, Healthy Eating Research, a national program of the Robert Wood Johnson Foundation, convened a panel of experts in the charitable food system, nutrition, and food policy fields to create clear, specific recommendations for evidence-based nutrition guidelines tailored to the unique needs and capacity of the charitable food system [47]. The intent of these recommendations is to improve the quality of foods in food banks and pantries in order to increase access to and promote healthier food choices.

Another concern of creating policies that stipulate the content of food donations is that such policies may reduce donations. However, in a 2012 survey of 74 food retailer respondents representing nearly 10,000 US supermarkets, the majority (89%) reported that concern about hunger was the primary incentive to donate food [45]. If that is true, and representative of other US food retailers, then policies that include nutritional requirements for donated foods and beverages may not substantially deter donations. Even so, the possible unintended consequences of such policies must be considered. Although not directly comparable, the 2018 report by MAZON and the Rudd Center found that, of the 196 food banks surveyed, the majority (85%) that had nutrition policies banning the distribution of some unhealthy beverages and snack foods reported that annual donations either increased or did not change [7]. Moreover, the food banks with formal nutrition policies and bans distributed twice as many fresh fruits and vegetables as unhealthy beverages and snack foods [7]. This suggests that prescriptive food banking policies hold potential to improve the nutritional quality of donated foods, while not negatively impacting the quantity of donations.

Looking beyond quantity and quality of donations, as Janet Poppendieck points out, there are numerous problems with reliance on charitable food aid that may still produce health and nutrition disparities, even if the nutritional quality of food donations improved [38]. For example, a mismatch between preferences and what is received, and the lack of dignity that many clients report feeling are virtually inherent when families depend on charitable food [38,48,49]. When the US emergency food system was first established, its clients were primarily families experiencing short term crisis and were unable to meet their food needs temporarily [50]. The past several years have seen a shift in which food pantry clients are chronically food insecure and regularly rely on the emergency food system [50]. An analysis of National Household Food Acquisition and Purchase Survey data found that charitable food assistance comprises 28% of total food at home acquisitions among clients [51]. Furthermore, among the families that use charitable food assistance and also participate in SNAP, approximately half (52%) exhausted their SNAP benefits within 10 days of receiving them [51]. Over two-thirds (68%) of those families relied on charitable food assistance, beginning on the 11th day after receiving SNAP benefits [51]. The emergency food system is meant to fill in gaps left by the federal safety net programs such as SNAP. However, the study by Fan et al. [51] suggests that the gap is substantial, and that families run out of SNAP benefits long before they will receive the next allotment. Families must then rely heavily on the private charitable food sector to fill the gap. Other recent work suggests that the amount families receive in SNAP benefits is insufficient [4,52]. To improve the food security and nutritional health of low-income families, the SNAP benefit formula should be modified to provide enough benefits to last throughout the month, which would also likely decrease reliance on the emergency food system [53].

Finally, acceptability and utilization of donated food are important dimensions of food insecurity [38,54]. Families relying on donated food may refuse or give away items that they do not know how to prepare or have the means to prepare [49]. If modifications to food donation policy result in less desirable food for those who use the emergency food system, then prescriptive donation policies could lead to some families forgoing emergency food entirely. Existing research suggests that families would prefer to receive more fresh meat and fresh produce [35,36,37]. Even so, food preferences are rarely universal, and some impoverished families do not have the resources necessary to prepare foods. Thus, some food banks in the United Kingdom provide “kettle boxes” that contain food products that can be prepared using only hot water [55,56]. For households without the option of hot water, food banks provide “cold boxes” that can be prepared without heating or adding hot water [56]. If prescriptive food donation policies restrict the donation of these items, then these policies could harm or deter the most vulnerable food bank clients. In order to mitigate negative unforeseen consequences of donation policy modifications, continued research in this area is warranted. For families who do have the means to prepare fresh foods, education programs focused on preparation of fresh, healthy food run by emergency feeding organizations may help ensure client use of this food [57]. However, education programs that are meant to be empowering may end up being patronizing [58]. Therefore, if organizations do offer food preparation education programs, the programs should be optional and not required for food bank clients.

In this review, we report on the presence or absence of a policy, which does not account for the implementation or enforcement of a policy. Many factors determine whether a policy achieves its intended outcome, including how well the policy ensures implementation [59]. Guidance documents may help, as they provide additional information about interpretation and implementation of the policy. For example, U.S. Department of Agriculture Memo on The Use of Share Tables in Child Nutrition Programs is key in disseminating information about Share Table programs [60]. This document clarified existing confusion among program administrators regarding whether donation of excess school foods was allowed. However, several key policies are nebulous in their implementation. For example, the Emerson Act, the central federal policy that protects the liability of food donors and the distributing organizations, does not fall under the auspices of any executive agency. As such, no executive agency oversees the Act’s implementation or interpretation, or raises awareness of the liability protections the Act provides [20]. The Federal Food Donation Act of 2008 illustrates another shortcoming in implementation. This Act requires that federal contracts over $25,000 for food provision, service, or sale, or private lease or rental of federal property for events with food include a provision that encourages surplus food donation. However, donations are only encouraged, not required or monitored [20]. Improving the oversight and implementation of existing policies is a potential avenue by which to promote healthy food and beverage donations.

This review has other limitations, including the dynamic nature of federal policy. The federal government may revise policies regularly, and as a result, this review may become outdated soon. Even so, the evolution of policy is a positive aspect of the US system. Updating and refining policies can better enable policies to achieve the desired outcomes. In addition, policy revision and the policymaking process allow public health practitioners and policy scholars to influence policymaking through advocacy. Strong nutrition-focused policies with comprehensive implementation details have the potential to increase food donations and improve the nutritional quality of donated foods. For example, in response to the COVID-19 pandemic, several policies have been passed recently that do or will likely affect food donations. First, via the Coronavirus Food Assistance Program (CFAP), the USDA is using $3 billion in funding provided in the Coronavirus Aid, Relief, and Economic Security (CARES) Act and the Families First Coronavirus Response Act (FFCRA) to purchase fresh produce, dairy, and meat to distribute to food banks and other nonprofit organizations [48,49,50]. In addition, the CARES Act and FFCRA provided $850 million for food banks’ administrative costs and USDA food purchases. These commodities will be distributed through existing programs, such as TEFAP [61,62,63,64]. Next, the CARES Act increased the 26 U.S.C. 170(3)(C) enhanced deduction from 15% of the taxpayer’s taxable income to 25% [61]. Together, these measures are meant to increase the capacity of the emergency food system to handle the heightened demand during the COVID-19 induced economic recession.

In taking into account these recent policies, none of the policies identified in this review explicitly encouraged the donation of healthy foods and beverages to food banks. This represents a missed opportunity, as the number of households relying on the charitable food system has increased substantially in light of the COVID-19 pandemic. Ensuring that families relying on the charitable food sector have adequate access to nutritious foods and beverages is one avenue through which to promote health and protect against diet-related chronic diseases.

## Figures and Tables

**Table 1:** Policy categories and example policies.

Category	Example
Liability protection	42 U.S.C. § 1791. (Bill Emerson Good Samaritan Food Donation Act) (c)(1) A person or gleaner shall not be subject to civil or criminal liability arising from the nature, age, packaging, or condition of apparently wholesome food or an apparently fit grocery product that the person or gleaner donates in good faith to a nonprofit organization for ultimate distribution to needy individuals.... (c)(2) A nonprofit organization shall not be subject to civil or criminal liability arising from the nature, age, packaging, or condition of apparently wholesome food or an apparently fit grocery product that the nonprofit organization received as a donation in good faith from a person or gleaner for ultimate distribution to needy individuals....
Tax incentive	26 U.S.C. § 170(3)(C). (Special rule for contributions of food inventory) (a)(1) There shall be allowed as a deduction any charitable contribution (as defined in subsection (c)) payment of which is made within the taxable year. (3)(C)(i) In the case of a charitable contribution of food from any trade or business of the taxpayer, this paragraph shall be applied only to food that is apparently wholesome food. (3)(C)(ii) The aggregate amount of such contributions for any taxable year may be taken into account under this section shall not exceed-...15 percent of taxable income....
Donation of certain food(s)	10 U.S.C. § 2485(f). (1) The Secretary of Defense may donate [commissary store food, mess food, meals ready-to-eat, and rations known as humanitarian daily rations] to any of the following entities: (A) A charitable nonprofit food bank that is designated by the Secretary of Defense or the Secretary of Health and Human Services as authorized to receive such donations.
Donation via schools	42 U.S.C. § 1758(l). (Richard B. Russell National School Lunch Act) Each school and local educational agency participating in the school lunch program under this Act may donate any food not consumed under such program to eligible local food banks or charitable organizations.
Grant program or fund	42 U.S.C. § 12572. An Opportunity Corps described in this paragraph may carry out activities such as-...carrying out a program that seeks to eliminate hunger in low-income communities and rural areas through service in projects … involving food banks, food pantries, and nonprofit organizations that provide food during emergencies;….
Government programs	42 U.S.C. § 1792. (Federal Food Donation Act of 2008) (a)...all contracts above $25,000 for the provision, service, or sale of food in the United States, or for the lease or rental of Federal property to a private entity for events at which food is provided in the United States, shall include a clause that- (1) encourages the donation of excess, apparently wholesome food to nonprofit organizations that provide assistance to food-insecure people in the United States;…

**Table 2:** Federal policies related to food donations.

Policy	Liability Protection	Tax Incentive	Donation of Certain Food(s)	Donation via Schools	Grant Program or Fund	Government Programs
**115 P.L. 334** Agriculture Improvement Act of 2018					x	x
**110 P.L. 246** Food, Conservation, and Energy Act of 2008			x		x	x
**113 P.L. 79** Agricultural Act of 2014			x	x		x
**7 U.S.C. § 2013(b)** Establishment of Supplemental Nutrition Assistance Program						x
**7 U.S.C. § 7502** Availability of CCC commodities						x
**7 U.S.C. § 7508** Emergency food assistance: Authorization and appropriations						x
**7 U.S.C. § 7511a** Emergency food program infrastructure grants					x	
**7 U.S.C. § 612c** (Section 3.2) Domestic food assistance programs						x
**42 U.S.C. § 1765** School lunch programs: Election to receive cash payments				x		
**42 U.S.C. § 1791** Bill Emerson Good Samaritan Food Donation Act	x					
**42 U.S.C. § 1792** Federal Food Donation Act of 2008						x
**42 U.S.C. § 12,572** National service programs eligible for program assistance					x	
**42 U.S.C. § 1758(l)** School lunch programs: Food donation program				x		
**42 U.S.C. § 1769(h)** School lunch programs: Pilot program for high-poverty schools				x		
**10 U.S.C. § 2485(f)** Commissary stores: operation			x			
**26 U.S.C. § 170(3)(C)** Internal revenue code: Charitable, etc., contributions and gifts		x				
**54 U.S.C. § 104,909** Wildlife management in parks			x			
**7 C.F.R. § 9** Coronavirus food assistance program						x
**7 C.F.R. §§ 252.1–252.7** National Commodity Processing Program						x
**7 C.F.R. §§ 251.1–251.10** The Emergency Food Assistance Program						x
**7 C.F.R. §§ 250.10–250.71** Donation of foods for use in the United States, its territories and possessions and areas under its jurisdiction						x
**7 C.F.R. §§ 240.1–240.11** Child nutrition programs: Cash in lieu of donated foods				x		
**7 C.F.R. §§ 235.1–235.12** Child nutrition programs: State administrative expense funds				x		
**7 C.F.R. § 220.8** School Breakfast Program: Meal requirements for breakfasts				x		
**7 C.F.R. §§ 210.4–210.8** Child nutrition programs: National School Lunch Program				x		
**7 C.F.R. § 210.2** National School Lunch Program: Definitions				x		
**7 C.F.R. § 210.19** National School Lunch Program: Additional responsibilities				x		
**7 C.F.R. §§ 210.9–210.16**. National School Lunch Program: Requirements for school food authority participation				x		
**7 C.F.R. § 170.12** What are the selection criteria for participation in the USDA Farmers Market?						x
**7 C.F.R. §§ 1430.300–1430.304** Dairy Product Donation Program						x
**7 C.F.R. §§ 1146.1–1146.210** Milk Donation Reimbursement Program						x
**48 C.F.R. §§ 26.400–26.404** Food donations to nonprofit organizations						x
**48 C.F.R. § 52.226–52.6** Promoting Excess Food Donation to Nonprofit Organizations						x
**50 C.F.R. §§ 679.2, 679.26** Fishers of the Exclusive Economic Zone off Alaska			x			
**50 C.F.R. § 635.32** Atlantic highly migratory species: Specifically authorized activities			x			
**45 C.F.R. § 2522.110** What types of programs are eligible to compete for AmeriCorps grants?					x	
**26 C.F.R. § 1.170A–1.4A** Special rule for the deduction of certain charitable contributions of inventory and other property.		x				
**1997 OLC 51** Preemptive Effect of the Bill Emerson Good Samaritan Food Donation Act	x					
**Number 1330.17** Department of Defense instruction: DoD Commissary Program			x			
**54 Comp. Gen. 192** In the matter of cash payments to states in lieu of donated commodities under the School Lunch Program				x		
**CARES Act HR 748** The Coronavirus Aid, Relief, and Economic Security Act		x				x
**FFCRA HR 6201** Families First Coronavirus Response Act						x
**Total**	2	3	7	12	5	19

CARES Act, Coronavirus Aid, Relief, and Economic Security Act; CFAP, Coronavirus Food Assistance Program; FFCRA, Families First Coronavirus Response Act; OLC; Office of Legal Counsel; USDA, United States Department of Agriculture. The x signifies that the policy falls within the category noted in the first row, e.g., the Agriculture Improvement Act of 2018 includes sections that we categorized as “grant program or fund” and “government program.”

## Supplementary Materials

The following are available online at https://www.mdpi.com/2072-6643/12/12/3764/s1, Table S1: Federal policies related to food donations.
