# Supplementary material for: Food Bank Donations in the United States: A Landscape Review of Federal Policies
Source: Nutrients. 2020 Dec 8;12(12):3764. doi: 10.3390/nu12123764 (PMC7762564; doi:10.3390/nu12123764)
Supplement: Supplementary file 1 [file nutrients-12-03764-s001.pdf]

Supplementary Table 1. Federal policies related to food donations

Policy	Excerpted policy text
115 P.L. 334[a]	• 7 U.S.C. § 9071. Milk donation program. (b) Program required; Purposes. [T]he Secretary shall establish and administer a milk donation program for the purposes of (1) encouraging the donation of eligible milk; (2) providing nutrition assistance to individuals in low income groups; and (3) reducing food waste.<br>• 7 U.S.C. § 7503(b). State plan. Section 202A(b) of the Emergency Food Assistance Act of 1983 (7 U.S.C. 7503(b)) is amended . . . (3) by adding at the end the following: "(5) at the option of the State agency, describe a plan of operation for 1 or more projects in partnership with 1 or more emergency feeding organizations located in the State to harvest, process, package, or transport donated commodities received under section 203D(d); and (6) describe a plan, which may include the use of a State advisory board established under subsection (c), that provides emergency feeding organizations or eligible recipient agencies within the State an opportunity to provide input on the commodity preferences and needs of the emergency feeding organization or eligible recipient agency."<br>• 7 U.S.C. § 7507. State and local supplementation of commodities. Section 203D of the Emergency Food Assistance Act of 1983 (7 U.S.C. 7507) is amended by adding at the end the following: ''(d) PROJECTS TO HARVEST, PROCESS, PACKAGE, OR TRANSPORT DONATED COMMODITIES.— (1) DEFINITION OF PROJECT.—In this subsection, the term 'project' means the harvesting, processing, packaging, or transportation of unharvested, unprocessed, or unpackaged commodities donated by agricultural producers, processors, or distributors for use by emergency feeding organizations under subsection (a). . . (3) PROJECT PURPOSES.—A State may only use Federal funds received under paragraph (2) for a project the purposes of which are— (A) to reduce food waste at the agricultural production, processing, or distribution level through the donation of food; (B) to provide food to individuals in need; and (C) to build relationships between agricultural producers, processors, and distributors and emergency feeding organizations through the donation of food. . . . Section 203D of the Emergency Food Assistance Act of 1983 (7 U.S.C. 7507), as amended by section 4018(c), is amended by adding at the end the following: ''(f) FOOD DONATION STANDARDS.— (1) DEFINITIONS.—In this subsection: (A) APPARENTLY WHOLESOME FOOD.—The term 'apparently wholesome food' has the meaning given the term in section 22(b) of the Child Nutrition Act of 1966 (42 U.S.C. 1791(b))."<br>• 7 U.S.C. § 2036d. Pilot projects to encourage the use of public-private partnerships committed to addressing food insecurity. (a) IN GENERAL.—The Secretary may, on application of eligible entities, approve not more than 10 pilot projects to support public-private partnerships that address food insecurity and poverty.<br>• 7 U.S.C. § 6924. Food Loss and Waste Reduction Liaison. (a) ESTABLISHMENT. The Secretary shall establish a Food Loss and Waste Reduction Liaison to coordinate Federal, State, local, and nongovernmental programs, and other efforts, to measure and reduce the incidence of food loss and waste in accordance with this section. (b) IN

	GENERAL.—The Food Loss and Waste Reduction Liaison shall . . . (4) raise awareness of the liability protections afforded under the Bill Emerson Good Samaritan Food Donation Act (42 U.S.C. 1791) to persons engaged in food loss and waste reduction and food recovery… ."<br>• 7 U.S.C. § 1627c.[b] Local agriculture market program. (a) PURPOSE.—The purpose of this section is to combine the purposes and coordinate the functions, as in effect on the day before the date of enactment of this Act, of— (1) the Farmers' Market and Local Food Promotion Program established under section 6 of the Farmer-to-Consumer Direct Marketing Act of 1976 (7 U.S.C. 3005); and (2) the value-added agricultural product market development grants under section 231(b) of the Agricultural Risk Protection Act of 2000 (7 U.S.C. 1632a(b)).
110 P.L. 246	• 7 U.S.C. § 2036.[c] Availability of commodities for emergency food assistance program. (a) PURCHASE OF COMMODITIES.—Section 27(a) of the Food and Nutrition Act of 2008 (7 U.S.C. 2036(a)) is amended by – (1) by striking ''(A) PURCHASE OF COMMODITIES'' and all that follows through ''$140,000,000 of'' and inserting the following: ''(a) PURCHASE OF COMMODITIES.—(1) IN GENERAL.—From amounts made available to carry out this Act, for each of the fiscal years 2008 through 2012, the Secretary shall purchase a dollar amount described in paragraph (2) of;<br>• 7 U.S.C. § 7511a. Emergency food program infrastructure grants. (a) DEFINITION OF ELIGIBLE ENTITY.—In this section, the term 'eligible entity' means an emergency feeding organization. (b) PROGRAM AUTHORIZED . . . (c) USE OF FUNDS.—An eligible entity shall use a grant received under this section for any fiscal year to carry out activities of the eligible entity, including—(1) the development and maintenance of a computerized system for the tracking of time-sensitive food products; (2) capital, infrastructure, and operating costs associated with the collection, storage, distribution, and transportation of time-sensitive and perishable food products; (3) improving the security and diversity of the emergency food distribution and recovery systems of the United States through the support of small or mid-size farms and ranches, fisheries, and aquaculture, and donations from local food producers and manufacturers to persons in need; (4) providing recovered foods to food banks and similar nonprofit emergency food providers to reduce hunger in the United States; (5) improving the identification of— (A) potential providers of donated foods; (B) potential nonprofit emergency food providers; and (C) persons in need of emergency food assistance in rural areas; and (6) constructing, expanding, or repairing a facility or equipment to support hunger relief agencies in the community.
113 P.L. 79	• 25 U.S.C. § 1685. Service of traditional foods in public facilities. (a) PURPOSES.—The purposes of this section are— (1) to provide access to traditional foods in food service programs; (2) to encourage increased consumption of traditional foods to decrease health disparities among Indians, particularly Alaska Natives; and (3) to provide alternative food options for food service programs.<br>• 7 U.S.C. § 6952. Multiagency task force. (a) IN GENERAL.—The Secretary shall establish, in the office of the Under Secretary for Food, Nutrition, and Consumer Services, a multiagency task force for the purpose of providing coordination and direction for commodity programs.

	• 2 U.S.C. § 1755b. Pulse crop products. (a) PURPOSE.—The purpose of this section is to encourage greater awareness and interest in the number and variety of pulse crop products available to schoolchildren, as recommended by most recent Dietary Guidelines for Americans published under section 301 of the National Nutrition Monitoring and Related Research Act of 1990 (7 U.S.C. 5341).
7 U.S.C. § 2013(b)	Food distribution program on Indian reservations.[d] (1) In general: Distribution of commodities, with or without the supplemental nutrition assistance program, shall be made whenever a request for concurrent or separate food program operations, respectively, is made by a tribal organization.
7 U.S.C. § 7502	(a) In general: Notwithstanding any other provision of law, in order to complement the domestic nutrition programs,...commodities acquired by the Commodity Credit Corporation that the Secretary of Agriculture (hereinafter referred to as the "Secretary") determines . . .are in excess of quantities needed to - (1) carry out other domestic donation programs, (2) meet other domestic obligations..., (3) meet international market development and food aid commitments, and (4) carry out the farm price and income stabilization purposes of the Agricultural Adjustment Act of 1938...shall be made available...without charge or credit...for food assistance.... (g)Donations to emergency feeding organizations: (1)Whenever commodities acquired by the Commodity Credit Corporation are made available for donation to domestic food programs in quantities that exceed Federal obligations, the Secretary shall give equal consideration to making donations of such commodities to emergency feeding organizations participating in the program authorized by this chapter as is given to other commodity recipient agencies, taking into account the types and amounts of commodities available and appropriate for distribution to these organizations. (2) In determining the commodities that will be made available to emergency feeding organizations under this chapter, the Secretary may distribute commodities that become available on a seasonal or irregular basis....
7 U.S.C. § 7508	The Emergency Food Assistance Program. Authorization and appropriations. (a) (1)There are authorized to be appropriated $100,000,000 for fiscal year 2008 and each fiscal year thereafter for the Secretary to make available to the States to pay for the direct and indirect costs of the States related to the processing, storage, transporting, and distributing to eligible recipient agencies of commodities provided by the Secretary under this chapter and commodities secured from other sources, including commodities secured by gleaning (as defined in section 111(a) of the Hunger Prevention Act of 1988 (7 U.S.C. 612c note; Public Law 100–435)) and donated wild game. . . (2)Each State shall make available to emergency feeding organizations in the State not less than 40 per centum of the funds provided as authorized in paragraph (1) that it has been allocated for a fiscal year, as necessary to pay for, or provide advance payments to cover, the direct expenses of the emergency feeding organizations for distributing commodities to needy persons, but only to the extent such expenses are actually so incurred by such organizations....
7 U.S.C. § 7511a	The Emergency Food Assistance Program. Emergency food program infrastructure grants. (a) Definition of eligible entity. In this section, the term "eligible entity" means an emergency feeding organization. (b)Program authorized:

	(1) In general. The Secretary shall use funds made available under subsection (d) to make grants to eligible entities to pay the costs of an activity described in subsection (c). (c) Use of funds. An eligible entity shall use a grant received under this section for any fiscal year to carry out activities of the eligible entity, including - …(3) improving the security and diversity of emergency food distribution and recovery systems of the United States through the support of small or mid-size farms and ranches, fisheries, and aquaculture, and donations from local food producers and manufacturers to persons in need; (4) providing recovered food to food banks...(5) improving the identification of (A) providers of donated food....
7 U.S.C. § 612c (Section 32)	Appropriation to encourage exportation and domestic consumption of agricultural products. There is appropriated for each fiscal year beginning with the fiscal year ending June 30, 1936 an amount equal to 30 per centum of the gross receipts from duties collected under the customs laws during the period January 1 to December 31, both inclusive, preceding the beginning of each such fiscal year. Such sums shall be maintained in a separate fund and shall be used by the Secretary of Agriculture only to . . .(2) encourage the domestic consumption of such commodities or products by diverting them, by the payment of benefits or indemnities or by other means, from the normal channels of trade and commerce or by increasing their utilization through benefits, indemnities, donations or by other means, among persons in low income groups as determined by the Secretary of Agriculture....
7 U.S.C. § 612c note	Commodity Distribution Program. (a) Notwithstanding any other provision of law, the Secretary may, during fiscal years 2008 through 2023, purchase and distribute sufficient agricultural commodities with funds appropriated from the general fund of the Treasury to maintain the traditional level of assistance for food assistance programs as are authorized by law.... Commodity Supplemental Food Program. (a) Grants Per Assigned Caseload Slot.—“(1)In general.— In carrying out the program under section 4 [set out as a note above] (referred to in this section as the ‘commodity supplemental food program’), for each of fiscal years 2008 through 2023, the Secretary shall provide to each State agency from funds made available to carry out that section (including any such funds remaining available from the preceding fiscal year), a grant per assigned caseload slot for administrative costs incurred by the State agency and local agencies in the State in operating the commodity supplemental food program..... SEC. 17. COMMODITY DONATIONS AND PROCESSING.“(a) In General.— Notwithstanding any other provision of law concerning commodity donations, any commodities acquired in the conduct of the operations of the Commodity Credit Corporation and any commodities acquired under section 32 of the Act of August 24, 1935 (7 U.S.C. 612c), to the extent that the commodities are in excess of the quantities of commodities that are essential to carry out other authorized activities of the Commodity Credit Corporation and the Secretary (including any quantity specifically reserved for a specific purpose), may be used for any program authorized to be carried out by the Secretary that involves the acquisition of commodities for use in a domestic feeding program, including any program conducted by the Secretary that provides commodities to individuals in cases of hardship. “(b)Programs.—A program described in subsection (a) includes a program authorized by— “(1) the Emergency Food Assistance Act of 1983 (7 U.S.C. 7501 et seq.); “(2) the Richard B. Russell National School Lunch Act (42 U.S.C. 1751 et seq.);.... Gleaning

	Clearinghouses. “(b) Establishment.—“(1) In general.—The Secretary of Agriculture (hereafter in this section referred to as the ‘Secretary’) is authorized to assist States and private nonprofit organizations in establishing Gleaning Clearinghouses (hereafter in this section referred to as a ‘Clearinghouse’)....
42 U.S.C. § 1765	National School Lunch Program. Election to receive cash payments. (a) Notwithstanding any other provision of law, where a State phased out its commodity distribution facilities prior to June 30, 1974, such State may, for purposes of the programs authorized by this chapter and the Child Nutrition Act of 1966 [42 U.S.C. 1771 et seq.], elect to receive cash payments in lieu of donated foods....
42 U.S.C. § 1791	Bill Emerson Good Samaritan Food Donation Act. (c) Liability for damages from donated food and grocery products: (1)Liability of person or gleaner: A person or gleaner shall not be subject to civil or criminal liability arising from the nature, age, packaging, or condition of apparently wholesome food or an apparently fit grocery product that the person or gleaner donates in good faith to a nonprofit organization for ultimate distribution to needy individuals. (2)Liability of nonprofit organization: A nonprofit organization shall not be subject to civil or criminal liability arising from the nature, age, packaging, or condition of apparently wholesome food or an apparently fit grocery product that the nonprofit organization received as a donation in good faith from a person or gleaner for ultimate distribution to needy individuals....
42 U.S.C. § 1792	Promoting Federal food donation. (a) In general. Not later than 180 days after June 20, 2008, the Federal Acquisition Regulation issued in accordance with section 1303 of title 41 shall be revised to provide that all contracts above $25,000 for the provision, service, or sale of food in the United States, or for the lease or rental of Federal property to a private entity for events at which food is provided in the United States, shall include a clause that (1)encourages the donation of excess, apparently wholesome food to nonprofit organizations that provide assistance to food-insecure people in the United States; and (2)states the terms and conditions described in subsection (b). (b)Terms and conditions: (1)Costs: In any case in which a contractor enters into a contract with an executive agency under which apparently wholesome food is donated to food-insecure people in the United States, the head of the executive agency shall not assume responsibility for the costs and logistics of collecting, transporting, maintaining the safety of, or distributing excess, apparently wholesome food to food-insecure people in the United States under this section. (2) Liability: An executive agency (including an executive agency that enters into a contract with a contractor) and any contractor making donations pursuant to this section shall be exempt from civil and criminal liability to the extent provided under section 1791 of this title.
42 U.S.C. § 12572	National service programs eligible for program assistance. (a) National service corps. The recipient of a grant under section 12571(a) of this title and a Federal agency operating or supporting a national service program under section 12571(b) of this title shall use a portion of the financial assistance or positions involved, directly or through subgrants to other entities, to support or carry out the following national service corps or programs, as full- or part-time corps or programs, to address unmet needs . . . (B) Activities: An Opportunity Corps described in this paragraph may carry out activities such as . . . (viii) carrying out a program that seeks to eliminate hunger in low-

	income communities and rural areas through service in projects (I) involving food banks, food pantries, and nonprofit organizations that provide food during emergencies . . . .
42 U.S.C. § 1758(l)	National School Lunch Program. Program requirements. (l) Food donation program: (1) In general: Each school and local educational agency participating in the school lunch program under this chapter may donate any food not consumed under such program to eligible local food banks or charitable organizations....
42 U.S.C. § 1769(h)	National School Lunch Program. Pilot projects. (B) Establishment: The Secretary shall carry out a pilot program under which the Secretary shall provide to nonprofit organizations or public entities in not more than 5 States grants to develop and run, through eligible programs, community gardens at eligible schools in the States . . . (D) Use of produce: Produce from a community garden provided a grant under this paragraph may be (i) used to supplement food provided at the eligible school; (ii)distributed to students to bring home to the families of the students; or (iii)donated to a local food bank or senior center nutrition program....
10 U.S.C. § 2485(f)	Commissary stores: operation. (f) Donation of Unusable Food: (1)The Secretary of Defense may donate food described in paragraph (2) to any of the following entities: (A)A charitable nonprofit food bank that is designated by the Secretary of Defense or the Secretary of Health and Human Services as authorized to receive such donations. (B)A State or local agency that is designated by the Secretary of Defense or the Secretary of Health and Human Services as authorized to receive such donations.... (2)Food that may be donated under this subsection is commissary store food, mess food, meals ready-to-eat (MREs), rations known as humanitarian daily rations (HDRs), and other food available to the Secretary of Defense that: (A)is certified as edible by appropriate food inspection technicians; (B)would otherwise be destroyed as unusable; and (C)in the case of commissary store food, is unmarketable and unsaleable....
26 U.S.C. § 170(3)(C)	Special rule for contributions of food inventory. (i) General rule. In the case of a charitable contribution of food from any trade or business of the taxpayer, this paragraph shall be applied - (I) without regard to whether the contribution is made by a C corporation, and (II) only to food that is apparently wholesome food. (ii) Limitation. The aggregate amount of such contributions for any taxable year...shall not exceed - … (II) in the case of a C corporation, 15 percent of taxable income (as defined in subsection (b)(2)(D))….
54 U.S.C. § 104909	Wildlife management in parks. (c) Donations.-The Secretary may authorize the donation and distribution of meat from wildlife management activities carried out under this section, including the donation and distribution to Indian Tribes, qualified volunteers, food banks, and other organizations that work to address hunger, in accordance with applicable health guidelines and such terms and conditions as the Secretary may require.
7 C.F.R. § 9	CORONAVIRUS FOOD ASSISTANCE PROGRAM. § 9.1. Applicability and administration. (a) This part specifies the eligibility requirements and payment calculations for the Coronavirus Food Assistance Program (CFAP). CFAP will provide payments with respect to commodities that have been significantly impacted by the effects of the COVID-19 outbreak. Payments will be made with respect to only commodities produced in the United States; commodities imported into the United States may not be used to determine any payment made under this part....

7 C.F.R. §§ 252.1 - .7	NATIONAL COMMODITY PROCESSING PROGRAM. § 252.1 Purpose and scope. (a) Purpose. This part provides a program whereby the Food and Nutrition Service (FNS) and private processors of food may enter into agreements under which the processor will process and distribute designated donated food to eligible recipient agencies. The intent of the program is to encourage private industry, acting in cooperation with the States and FNS, to develop new markets in which donated food may be utilized. It is expected that the processors will use their marketing abilities to encourage eligible recipient agencies to participate in the program....
7 C.F.R. §§ 251.1 - .10	THE EMERGENCY FOOD ASSISTANCE PROGRAM. § 251.1. General purpose and scope. This part announces the policies and prescribes the regulations necessary to carry out certain provisions of the Emergency Food Assistance Act of 1983, (7 U.S.C. 612c). . . . § 251.6. Distribution plan. (5) At the option of the State agency, a plan of operation for one or more projects in partnership with one or more emergency feeding organizations located in the State to harvest, process, package, or transport donated commodities received under section 203D(d) of the Emergency Food Assistance Act of 1983. The plan must include all items listed in paragraphs (a)(5)(i) through (iv) of this section: (i) A list of emergency feeding organizations within the State that will operate the project in partnership with the State agency. (ii) A list of any State agencies that will operate the project as a part of a cooperative agreement. (iii) A description of the purpose of the Farm to Food Bank Project that includes how the Project will: (A) Reduce food waste at the agricultural production, processing, or distribution level through the donation of food; (B) Provide food to individuals in need; and (C) Build relationships between agricultural producers, processors, and distributors and emergency feeding organizations through the donation of food. . . . § 251.9. Matching of funds. (a) State matching requirement. The State must provide a cash or in-kind contribution equal to the amount of TEFAP administrative funds received under § 251.8 and retained by the State agency for State-level costs or made available by the State agency directly to eligible recipient agencies that are not emergency feeding organizations as defined in § 251.3(e). . . . § 251.10. Miscellaneous provisions. (j) Projects to harvest, process, package, or transport donated commodities: (1) Definition of project. These projects, also known as Farm to Food Bank Projects, are defined as the harvesting, processing, packaging, or transportation of unharvested, unprocessed, or unpackaged commodities donated by agricultural producers, processors, or distributors for use by emergency feeding organizations under section 203D of the Emergency Food Assistance Act of 1983....
7 C.F.R. §§ 250.10 - .71[e]	DONATION OF FOODS FOR USE IN THE UNITED STATES, ITS TERRITORIES AND POSSESSIONS AND AREAS UNDER ITS JURISDICTION. Subpart B. Delivery, Distribution, and Control of Donated Foods. § 250.10. Availability and ordering of donated foods. (a) Ordering donated foods. The distributing agency must utilize a request-driven ordering system in submitting orders for donated foods to FNS. As part of such system, the distributing agency must provide recipient agencies with the opportunity to submit input, on at least an annual basis, in determining the donated foods from the full list that are made available to them for ordering. Based on the input received, the distributing agency must ensure that the types and forms of donated foods that recipient

	agencies may best utilize are made available to them for ordering. The distributing agency must also ensure that donated foods are ordered and distributed only in amounts that may be utilized efficiently and without waste. (b) Provision of information on donated foods. The distributing agency must provide recipient agencies, at their request, information that will assist them in ordering or utilization of donated foods, including information provided by USDA. Information provided to recipient agencies must include: (1) The types and quantities of donated foods that they may order; (2) Donated food specifications and nutritional value; and (3) Procedures for the disposition of donated foods that are out-of-condition or that are subject to a food recall....
7 C.F.R. §§ 240.1 - .11[d]	CHILD NUTRITION PROGRAMS. § 240.1. General purpose and scope. (a) Each school year the Department programs agricultural commodities and other foods to States for delivery to program and commodity schools, nonresidential child care institutions, and service institutions pursuant to the regulations governing the donation of foods for use in the United States, its territories and possessions and areas under its jurisdiction (7 CFR part 250). (b) Section 6(b) of the Act requires that not later than June 1 of each school year, the Secretary shall make an estimate of the value of the agricultural commodities and other foods that will be delivered during that school year for use in lunch programs by schools participating in the National School Lunch Program (7 CFR part 210). If this estimate is less than the total level of assistance authorized under section 6(e) of the Act the Secretary shall pay to the State administering agency not later than July 1 of that school year, an amount of funds equal to the difference between the value of donated foods as then programmed for that school year and the total level of assistance authorized under such section....(g) Section 16 of the Act provides that a State which has phased out its food distribution facilities prior to June 30, 1974, may elect to receive cash payments in lieu of donated foods....
7 C.F.R. §§ 235.1 - .12[d]	CHILD NUTRITION PROGRAMS. § 235.1. General purpose and scope. This part announces the policies and prescribes the regulations necessary to carry out the provisions of section 7 of the Child Nutrition Act of 1966, as amended. It prescribes the methods for making payments of funds to State agencies for use for administrative expenses incurred in supervising and giving technical assistance in connection with activities undertaken by them under the National School Lunch Program (7 CFR part 210), the Special Milk Program (7 CFR part 215), the School Breakfast Program (7 CFR part 220), the Child and Adult Care Food Program (7 CFR part 226) and the Food Distribution Program (7 CFR part 250)....
7 C.F.R. § 220.8	CHILD NUTRITION PROGRAMS. § 220.8. Meal requirements for breakfasts. (a) General requirements. This section contains the meal requirements applicable to school breakfasts for students in grades K through 12, and for children under the age of 5. In general, school food authorities must ensure that participating schools provide nutritious, well-balanced, and age-appropriate breakfasts to all the children they serve to improve their diet and safeguard their health....
7 C.F.R. §§ 210.4 - .8	NATIONAL SCHOOL LUNCH PROGRAM. § 210.4. Cash and donated food assistance to States. (a) General. To the extent funds are available, FNS will make cash assistance available in accordance with the provisions of this section to each State agency for lunches and meal supplements served to children under the National School Lunch

	and Commodity School Programs. To the extent donated foods are available, FNS will provide donated food assistance to distributing agencies for each lunch served in accordance with the provisions of this part and part 250 of this chapter....
7 C.F.R. § 210.2	NATIONAL SCHOOL LUNCH PROGRAM. Subpart A. General. Definitions: . . . Commodity School Program means the Program under which participating schools operate a nonprofit lunch program in accordance with this part and receive donated food assistance in lieu of general cash assistance. Schools participating in the Commodity School Program shall also receive special cash and donated food assistance in accordance with § 210.4(c)….
7 C.F.R. § 210.19	NATIONAL SCHOOL LUNCH PROGRAM. Subpart D. Requirements for State Agency Participation. Additional responsibilities. ...(b) Donated food distribution information. Information on schools eligible to receive donated foods available under section 6 of the National School Lunch Act (42 U.S.C. 1755) shall be prepared each year by the State agency with accompanying information on the average daily number of lunches to be served in such schools. This information shall be prepared as early as practicable each school year and forwarded no later than September 1 to the Distributing agency. The State agency shall be responsible for promptly revising the information to reflect additions or deletions of eligible schools, and for providing such adjustments in participation as are determined necessary by the State agency. Schools shall be consulted by the Distributing agency with respect to the needs of such schools relating to the manner of selection and distribution of commodity assistance....
7 C.F.R. §§ 210.9 - .16.	NATIONAL SCHOOL LUNCH PROGRAM. § 210.10. Meal requirements for lunches and requirements for afterschool snacks. (a) General requirements—(1) General nutrition requirements. Schools must offer nutritious, well-balanced, and age-appropriate meals to all the children they serve to improve their diets and safeguard their health. . . . (i) Requirements for lunch. School lunches offered to children age 5 or older must meet, at a minimum, the meal requirements in paragraph (b) of this section. Schools must follow a food-based menu planning approach and produce enough food to offer each child the quantities specified in the meal pattern established in paragraph (c) of this section for each age/grade group served in the school. In addition, school lunches must meet the dietary specifications in paragraph (f) of this section. Schools offering lunches to children ages 1 through 4 and infants must meet the meal pattern requirements in paragraphs (p) and (q), as applicable, of this section. Schools must make potable water available and accessible without restriction to children at no charge in the place(s) where lunches are served during the meal service. (ii) Requirements for afterschool snacks. Schools offering afterschool snacks in afterschool care programs must meet the meal pattern requirements in paragraph (o) of this section. Schools must plan and produce enough food to offer each child the minimum quantities under the meal pattern in paragraph (o) of this section.
7 C.F.R. § 170.12	What are the selection criteria for participation in the USDA Farmers Market?<br>The selection criteria are designed to ensure a consistently high level of quality and diverse products are available at the market, while operating in the constraints of space available at the market site. The criteria are: . . . (c) Willingness to Glean. Participants should commit to supporting the USDA food gleaning/food recovery initiative.

	This commitment requires farmers and vendors to donate surplus food and food products at the end of each market day to a local nonprofit organization identified by USDA. Questions about tax deductions for gleaning should be referred to the Internal Revenue Service or a tax advisor. Receipts for donated foods may be obtained from the receiving nonprofit organization.
7 C.F.R. §§ 1430.300 - .304	COMMODITY CREDIT CORPORATION, DEPARTMENT OF AGRICULTURE. § 1430.300. Administration, purpose, and funding. (a) The regulations in this subpart apply for the Dairy Product Donation Program (DPDP). DPDP is authorized by section 1431 of the Agricultural Act of 2014 (Pub. L. 113-79, 7 U.S.C. 9071). (b) DPDP is designed to address low dairy producer margins, through periodic purchases of dairy products, as specified in this subpart. Dairy products purchased for DPDP will be used to provide nutritional assistance to members of low-income groups. . . . § 1430.304. Distribution of DPDP purchased products: (a) Purchased products will be distributed to private and public nonprofit organizations eligible to receive donated foods for distribution to low-income groups through FNS' food distribution programs as specified in FNS program regulations and the requirements in 7 CFR part 250. (b) Public and private nonprofit organizations receiving donated dairy products under this section will be responsible for the proper handling and distribution of such products in accordance with FNS program regulations, 7 CFR part 250, and FNS guidance and instructions. (c) A private or nonprofit organization agency receiving donated products under this section which improperly distributes or uses such product or causes loss of or damage to such product, will be subject to recovery of losses or other corrective action in accordance with FNS program regulations, 7 CFR part 250.
7 C.F.R. §§ 1146.1 - .210	MILK DONATION REIMBURSEMENT PROGRAM. § 1146.1. Program eligibility. An eligible dairy organization must be a member of a participating partnership pursuant to § 1146.1 to be eligible to receive reimbursements for qualified expenses related to voluntary fluid milk donations, subject to the requirements and limitations specified in §§ 1146.102 and 1146.104. §1146.106. Reimbursement claims: (a) In order for the eligible dairy organization partner to receive reimbursements for qualified expenses pursuant to § 1146.108, the participating partnership must submit a report and appropriate supporting documentation to Agricultural Marketing Service. . . . (2) Appropriate documentation to support the report required in paragraph (a)(1) of this section may include, but is not limited to, copies of processing records, shipping records, bills of lading, warehouse receipts, distribution records, or other documents that demonstrate the reported amount of eligible milk was processed, donated, and distributed in accordance to the approved Milk Donation and Distribution Plan and as reported in the eligible dairy organization's report.
48 C.F.R. §§ 26.400 - .404	§ 26.400. This section implements the Federal Food Donation Act of 2008 (42 U.S.C. 1792).§ 26.402. Policy. The Government encourages executive agencies and their contractors, to the maximum extent practicable and safe, to donate excess apparently wholesome food to nonprofit organizations that provide assistance to food-insecure people in the United States. §26.403. Procedures. (a) ...an executive agency shall comply with the following: (1) Encourage donations. In the applicable contracts stated at section 26.404, encourage contractors, to the maximum

	extent practicable and safe, to donate apparently wholesome excess food to nonprofit organizations that provide assistance to food-insecure people in the United States. (2) Costs. (I) In any case in which a contractor enters into a contract with an executive agency under which apparently wholesome food is donated to food-insecure people...the head of the executive agency shall not assume responsibility for the costs and logistics [of donation]…(ii) The Government will not reimburse any costs incurred by the contractor against this contract or any other contract for the donation of Federal excess foods...
48 C.F.R. § 52.226-6	Promoting Excess Food Donation to Nonprofit Organizations. . . . (b) In accordance with the Federal Food Donation Act of 2008 (42 U.S.C. 1792), the Contractor is encouraged, to the maximum extent practicable and safe, to donate excess, apparently wholesome food to nonprofit organizations that provide assistance to food-insecure people in the United States.
50 C.F.R. §§ 679.2, 679.26	§ 679.2. Definitions. Authorized distributor means a tax-exempt organization authorized by National Marine Fisheries Service to coordinate the processing, storage, transportation, and distribution of salmon taken as incidental catch in the groundfish trawl fisheries to tax-exempt hunger relief agencies, food bank networks, and food bank distributors (see § 679.26, Prohibited Species Donation Program). . . . §679.26. Prohibited Species Donation Program. (a) Authorized species. The PSD program applies only to the following species: (1) Salmon. (2) Halibut delivered by catcher vessels using trawl gear to shoreside processors and stationary floating processors. (b) Authorized distributors: (1) Application. An applicant seeking to become an authorized distributor must provide the Regional Administrator with the following information: (i) Proof of the applicant's tax-exempt status. (ii) A description of the arrangements for processing, shipping, storing, and transporting donated fish and an estimate of the associated costs. (iii) A statement describing the applicant's expertise in providing for the distribution of food product from remote Alaskan locations to hunger relief agencies, food bank networks, or food bank distributors, including arrangements for transportation, distribution costs, and product quality control....
50 C.F.R. § 635.32	Specifically authorized activities. (a) General. (1) Consistent with the provisions of § 600.745 of this chapter, except as indicated in this section, NMFS may authorize activities otherwise prohibited by the regulations contained in this part for the conduct of scientific research, the acquisition of information and data, the enhancement of safety at sea, the purpose of collecting animals for public education or display, the investigation of bycatch, economic discard and regulatory discard, or for chartering arrangements. (2) Activities subject to the provisions of this section include, but are not limited to: scientific research resulting in, or likely to result in, the take, harvest, or incidental mortality of Atlantic HMS; exempted fishing and educational activities; programs under which regulated species retained in contravention to otherwise applicable regulations may be donated through approved food bank networks; or chartering arrangements....
45 C.F.R. § 2522.110	What types of programs are eligible to compete for AmeriCorps grants? Types of programs eligible to compete for AmeriCorps grants include the following: . . . (b) Specialized service programs.... (2) A program that seeks to eliminate hunger in communities and rural areas through service in projects -

	(i) Involving food banks, food pantries, and nonprofit organizations that provide food during emergencies; (ii) Involving the gleaning of prepared and unprepared food that would otherwise be discarded as unusable so that the usable portion of such food may be donated to food banks, food pantries, and other nonprofit organizations;...
26 C.F.R. § 1.170A-4A	Special rule for the deduction of certain charitable contributions of inventory and other property. (a) Introduction. Section 170(e)(3) provides a special rule for the deduction of certain qualified contributions of inventory and certain other property. . .the property must be used for the care of the ill, needy, or infants....Under paragraph (b)(5) of this section, the contributed property must conform to any applicable provisions of the Federal Food, Drug, and Cosmetic Act (as amended), and the regulations thereunder, at the date of contribution and for the immediately preceding 180 days. . . . (b) Qualified contributions. . . . (2) Restrictions on use of contributed property. . . (3) Restrictions on Transfer of contributed property - (i) In general. Except as otherwise provided in subdivision (ii) of this paragraph (b)(3), a contribution will not qualify...if the donee-organization...requires or receives any money, property, or services for the transfer or use of property contributed under section 170(e)(3)….(ii) Exception. A contribution may qualify under this section if the donee-organization charges a fee to another organization in connection with its transfer of the donated property, if: (A) The fee is small or nominal in relation to the value of the transferred property and is not determined by this value; and (B) The fee is designed to reimburse the donee-organization for its administrative, warehousing, or other similar costs. For example, if a charitable organization (such as a food bank) accepts surplus food to distribute to other charities which give the food to needy persons, a small fee may be charged to cover administrative, warehousing, and other similar costs. This fee may be charged on the basis of the total number of pounds of food distributed to the transferee charity but not on the basis of the value of the food distributed. The provisions of this subdivision (ii) do not apply to a transfer of donated property directly from an organization to ill or needy individuals, or infants.
1997 OLC 51	Preemptive Effect of the Bill Emerson Good Samaritan Food Donation Act. The Emerson Act preempts state “good samaritan” statutes that provide less protection from civil and criminal liability arising from food donated in good faith for distribution to the needy than the Act provides.
Number 1330.17	Armed Services Commissary Operations. PURPOSE. In accordance with the authority in DoD Directive (DoDD) 5124.02 (Reference (a)), this instruction reissues DoD Instruction (DoDI) 1330.17 (Reference (b)) to establish policy, assign responsibilities, and prescribe procedures for operating the DoD commissary program. . . . CHARITABLE ORGANIZATIONS: a. Donations: (1) Pursuant to section 2485(f) of Reference (c), DeCA may donate unmarketable, unsaleable, but edible food to:<br>(a) A nonprofit, charitable food bank exempt from federal income taxation pursuant to section 501(c)(3) of Title 26, U.S.C. (Reference (t)) and designated by the ASD(R&FM) or the Secretary of Health and Human Services to receive such donations; (b) A State or local agency authorized to receive donations from the U.S. Department of Agriculture Food and Nutrition Service Food Distribution Division;...

54 Comp. Gen. 192	IN THE MATTER OF CASH PAYMENTS TO SCHOOLS IN LIEU OF DONATED COMMODITIES UNDER THE SCHOOL LUNCH PROGRAM. Department of Agriculture has authority under National School Lunch Act, amended by Public Law 93-326, to make cash payments to states for School Lunch Program in lieu of donating any commodities, where distribution of donated commodities is not possible, since such authority is expressly recognized and affirmed in Conference Report on Public Law 93-326 and is otherwise consistent with statutory language and legislative history.
CARES Act HR 748	§ 2205. MODIFICATION OF LIMITATIONS ON CHARITABLE CONTRIBUTIONS DURING 2020. (b) INCREASE IN LIMITS ON CONTRIBUTIONS OF FOOD INVENTORY.—In the case of any charitable contribution of food during 2020 to which section 170(e)(3)(C) of the Internal Revenue Code of 1986 applies, subclauses (I) and (II) of clause (ii) thereof shall each be applied by substituting ''25 percent'' for ''15 percent.''<br>(c) EFFECTIVE DATE.—This section shall apply to taxable years ending after December 31, 2019. . . .<br>§ 6002. For an additional amount for ''Commodity Assistance Program,'' $450,000,000, to remain available through September 30, 2021, to prevent, prepare for, and respond to coronavirus, domestically or internationally, for the emergency food assistance program as authorized by section 27(a) of the Food and Nutrition Act of 2008 (7 U.S.C. 2036(a)) and section 204(a)(1) of the Emergency Food Assistance Act of 1983 (7 U.S.C. 7508(a)(1)): Provided, That of the funds made available, the Secretary may use up to $150,000,000 for costs associated with the distribution of commodities: Provided further, That such amount is designated by the Congress as being for an emergency requirement pursuant to section 251(b)(2)(A)(i) of the Balanced Budget and Emergency Deficit Control Act of 1985.
FFCRA HR 6201	§ 3. For an additional amount for the ''Commodity Assistance Program'' for the emergency food assistance program as authorized by section 27(a) of the Food and Nutrition Act of 2008 (7 U.S.C. 2036(a)) and section 204(a)(1) of the Emergency Food Assistance Act of 1983 (7 U.S.C. 7508(a)(1)), $400,000,000, to remain available through September 30, 2021: Provided, That of the funds made available, the Secretary may use up to $100,000,000 for costs associated with the distribution of commodities: Provided further, That such amount is designated by the Congress as being for an emergency requirement pursuant to section 251(b)(2)(A)(i) of the Balanced Budget and Emergency Deficit Control Act of 1985.

[a] 115 P.L. 334, 110 P.L. 246, and 113 P.L. 79 are the 2018, 2008, and 2014 Farm Bills, respectively. In some cases, these bills are the legislation in which a bullet-pointed statute or provision of a statute was first introduced (e.g., 115 P.L. 334 introduced 7 U.S.C. § 9071). However, in other cases, these Farm Bills amended an existing statute or provision of a statute that did not show up in our search independently of the Farm Bill-the Farm Bill made us aware of the laws' existence during our scan and therefore, we included them instead of the statutes themselves.

[b] This statute created the Local Agriculture Market Program (LAMP) that serves as an umbrella for several existing programs, including the Local Food Promotion Program, a program though which food banks can receive grants to work with local food producers.
[c] The 2018 Farm Bill updated the fiscal years in this statute but retained the funding structure.
[d] The Food Distribution Program on Indian Reservations (FDPIR) is a provision in Supplemental Nutrition Assistance Program statute. The program distributes USDA commodities to tribal communities, often where there are no SNAP offices or SNAP retailers. Unlike other Federal commodities programs, FDPIR offerings include traditional foods such as bison meat and wild rice, and locally- or regionally-grown foods.
[e] This C.F.R Subpart includes more relevant sections than it is possible to include in this Table.
